# Effect of lithium disilicate ceramic thickness, shade and translucency on transmitted irradiance and knoop microhardness of a light cured luting resin cement

**DOI:** 10.1007/s10856-021-06562-2

**Published:** 2021-07-31

**Authors:** Lincoln Pires Silva Borges, Gilberto Antônio Borges, Américo Bortolazzo Correr, Jeffrey A. Platt, Sidney Kina, Lourenço Correr-Sobrinho, Ana Rosa Costa

**Affiliations:** 1 Department of Restorative Dentistry, Dental Materials Division, Piracicaba Dental School, State University of Campinas – UNICAMP, Piracicaba, SP Brazil; 2 Department of Dental Materials and Restorative Dentistry, University of Uberaba, Uberaba, MG Brazil; 3 Associate Professor of Dental Materials, Indiana University School of Dentistry, Indianapolis, IN USA; 4 Associate Professor, Kina Essential Balance Institute, Maringá, PR Brazil

## Abstract

This in vitro study evaluates the influence of pressed lithium disilicate thickness, shade and translucency on the transmitted irradiance and the Knoop microhardness (KHN) of a light-cured resin cement at two depths. One hundred and thirty-five ceramic discs of IPS e.max Press (Ivoclar Vivadent) were fabricated and divided into twenty-seven groups (*n* = 5) according to the association between translucency: HT (hight translucency), LT (low translucency), and MO (medium opacity); shade: BL2, A1 and A3.5; and thickness: 0.5 mm, 1.5 mm, and 2.0 mm. One side of each ceramic disc was finished, polished and glazed. The irradiance (mW/cm²) of a multiwave LED light curing unit (Valo, Ultradent) was evaluated with a potentiometer (Ophir 10ª-V2-SH, Ophir Optronics) without (control group) or with interposition of ceramic samples. The microhardness of Variolink Esthetic LC resin cement (Ivoclar Vivadent) was evaluated after 24 h at two depths (100 μm and 700 μm). Data were submitted to ANOVA followed by Tukey’s test (*α* = 0.05). Irradiance and KHN were significantly influenced by ceramic thickness (*p* < 0.0001), shade (*p* < 0.001), translucency (*p* < 0.0001) and depth (*p* < 0.0001). Conclusions: the interposition of increasing ceramic thicknesses significantly reduced the irradiance and microhardness of resin cement. Increased depth in the resin cement showed significantly reduced microhardness for all studied groups. Increased ceramic opacity reduced the KHN of the resin cement at both depths for all ceramic thicknesses and shades.

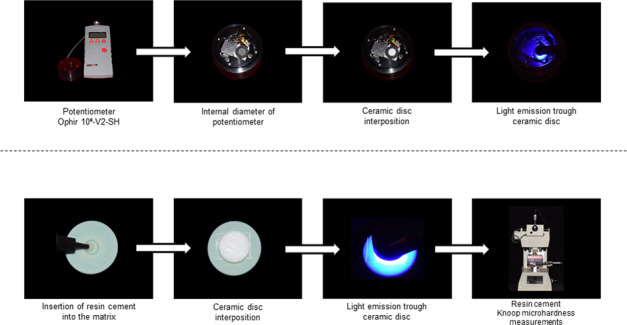

## Introduction

Dental ceramics are used in dentistry to replace lost portions of teeth. They provide significant mechanical properties that contribute to the success of dental restorations. Their wide range of indications is due to desirable characteristics, such as good biocompatibility, color stability, chemical durability, improved flexural strength, lower thermal conductivity, radiopacity, and ability to mimic function and esthetics [1–5]. Lithium disilicate reinforced by orthophosphate crystals in a vitreous, glassy matrix can be used in a pressed or milled form. It is reported to have excellent clinical performance and mechanical properties [6–9]. According to the manufacturer of IPS e.max Press, a pressed lithium disilicate ceramic (Ivoclar Vivadent, Schaan, Liechtenstein), several translucencies and shade ingots are available.

In order to enhance ceramic mechanical properties and clinical longevity, the cementation procedure is an important factor and depends on the ceramic type and the ceramic surface preparation [4, 10]. The lithium disilicate glass ceramic is acid-sensitive and undergoes surface degradation when subjected to hydrofluoric acid that results in an increased contact surface area interaction between the ceramic and luting agent [4, 11]. After silane application, a resin cement is used for luting. These cements are available as chemical, photo or dual activated systems [12]. Adequate light energy must reach the resin cement to ensure optimal polymerization [13].

Inadequate polymerization of the resin cement results in poor mechanical properties, higher solubility and decreased color stability [14, 15]. Many studies have shown that ceramics attenuate light and may compromise the photo-activation of the resin cement [13, 16, 17]. The ceramic translucency, thickness, shade and crystalline structure are factors that impact the amount of attenuation [16–23]. Decreased light transmittance decreases the total energy that reaches the composite resin, resulting in inadequate polymerization [13].

When placing restorations, it is important to use techniques that optimize physical properties, clinical performance and longevity. Previous reports [13–16, 19–23] assessed only one or two of the above mentioned ceramic factors related to the physical properties of resin cements activated by a conventional camphorquinone/tertiary amine reaction. More understanding is needed about the influence of shade, thickness and translucency on characteristics such as the light transmitted through pressed lithium disilicate ceramic and Knoop microhardness of a resin cement formulated with Ivocerin^®^ (Ivoclar Vivadent, Schaan, Liechtenstein) a germanium based photoinitiator associated with thiocarbamide used for better long term color stability.

The aim of this study was to evaluate the influence of shade, thickness, and translucency of a pressable lithium disilicate ceramic on the transmitted irradiance through the ceramic and Knoop microhardness of a resin cement. The hypotheses tested were: (1) The transmitted irradiance is not impacted by different shades, translucencies and thicknesses of the ceramic; and (2) The Knoop microhardness of a resin cement is not impacted by different shades, translucencies, thicknesses and depths of the ceramic.

## Materials and methods

### Preparation of samples

**Fig. 1:**
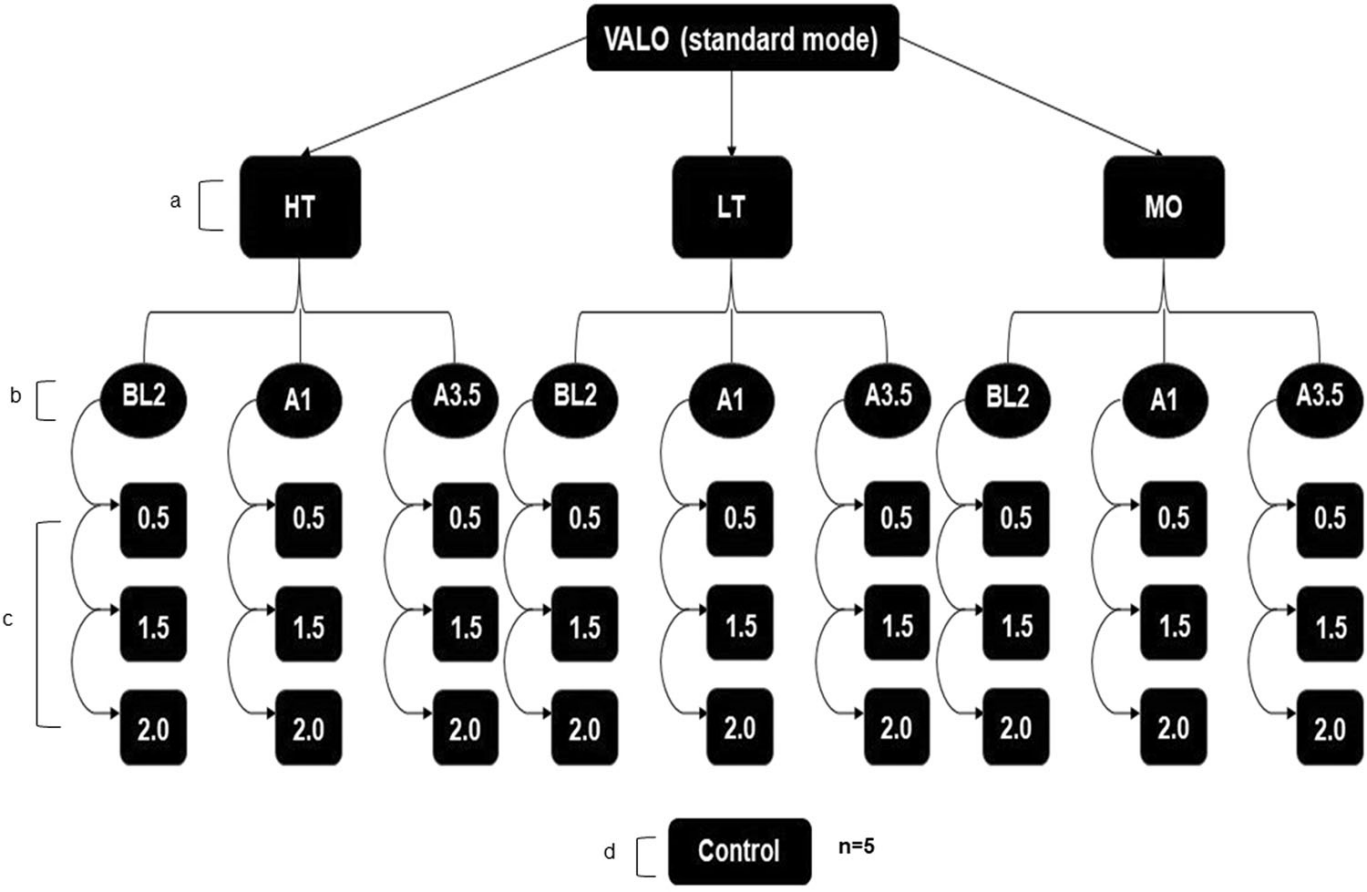
Organization chart of the study groups. **a** Translucencies: HT (high translucency), LT (low translucency), and MO (medium opacity). **b** Shades: A3.5, A1 and BL2. **c** Thicknesses: 0.5 mm, 1.5 mm, and 2.0 mm. **d** Control group: without interposition of ceramic

One hundred and thirty-five ceramic discs of IPS e.max Press (Ivoclar Vivadent) were fabricated and divided into twenty-seven groups (*n* = 5) according to the association between translucencies: HT (high translucency), LT (low translucency), MO (medium opacity), shades: BL2, A1 and A3.5 (due to different shade nomenclature provided by manufacturer for MO, shades 0, 1, and 3 were used as matches for BL2, A1 and A3.5, respectively), and thickness: 0.5 mm, 1.5 mm, and 2.0 mm (Fig. 1). Acrylic resin cylinders (Duralay, Reliance Dental MFG Company, Illinois, USA) of 12 mm diameter were cut into discs in the thicknesses of 0.7 mm, 1.7 mm, and 2.2 mm using a 0.3 mm-diamond blade (Buehler, Lake Bluff, IL, USA) mounted in a slow speed IsoMet machine (Buehler, Lake Bluff, IL, USA). Each disc was attached to a sprue former and fixed on a flask base that was coupled to a silicone cylinder. They were invested using a phosphate-bonded investment material (IPS PressVest Speed, Ivoclar Vivadent, Schaan, Liechtenstein) and eliminated in an automatic furnace (Vulcan A- 550, Degussa-Ney, Yucaipa, CA, USA) at a temperature of 850 °C for 60 min. IPS e.max Press ceramic ingots were heated to 920 °C and pressed into the investment molds in an automatic press furnace (EP 600, Ivoclar Vivadent).

After recovery from the investment, one side of each ceramic disc was finished and polished with stones (DT01, DT08 diamond stone – Diaturbo, Ribeirão Preto, Brazil) and a rubber point (DT-H14Dmf – EVE, Keltern, Germany) to simulate the fabrication process of an indirect ceramic restoration. The other side of the disc was not polished. All samples were ultrasonically cleaned in deionized water (Ultrasonic Cleaner 1440D, Cristófoli, Campo Mourão, Brazil) for 10 min and dried with compressed air. The final disc thicknesses (0.5 mm, 1.5 mm, and 2.0 mm) were confirmed with a digital caliper (Mitutoyo Corporation, Tokyo, Japan) having an accuracy of 0.01 mm. Two layers of a glaze paste (IPS e.max Ceram Glaze, Ivoclar Vivadent, Schaan, Liechtenstein) were applied only to the polished ceramic surface with movements in a single direction. Each glaze layer was sintered (P710 Programat, Ivoclar Vivadent, Schaan, Liechtenstein) for 15 min and allowed to cool.

### Light irradiance analysis (mW/cm²)

The irradiance of a light-emitting diode (LED) device (Valo Cordless, standard mode, Ultradent Inc., South Jordan, UT, USA) was evaluated through the ceramic samples. The LED device was secured to allow positioning the light guide tip perpendicular to the material surface. Measurements (mW) were obtained with a potentiometer (Ophir 10ª-V2-SH, Ophir Optronics, Har- Hotzvim, Jerusalem, Israel) coupled to a microprocessor (NOVA, Ophir Optronics), under controlled conditions of humidity (50 ± 10%) and temperature (23 ± 2 °C). A black Propylene matrix (Marpax, São Paulo, SP, Brazil) was used to limit the area of the potentiometer sensor to a diameter of 12 mm. Three readings were made after 60 s for each sample with the light guide tip in contact with the ceramic glazed surface of the ceramic discs. For the control group, five readings were performed using the same procedure but without the interposition of ceramic. The irradiance (mW/cm^2^) was determined by dividing mW by the area of the output light guide for the LED device (cm^2^).

Light Irradiance data after the exploratory analysis were submitted to analysis of variance (ANOVA) in a 3 × 3 × 3 + 1 (translucency × shade × thickness + control) factorial scheme. Appropriate post-hoc pair wise comparisons of means were tested using Tukey’s test (SAS Institute Inc., Cary, NC, USA, version 9.3). All statistical tests were performed at *α* = 0.05.

### Knoop microhardness test

Five samples of Variolink Esthetic LC warm (Ivoclar Vivadent) resin cement were fabricated for each study group. The resin cement was dispensed directly into an elastomeric mold (Express putty; 3 M ESPE, St. Paul, MN, USA) with a 5 mm diameter and 1 mm thickness. Over this was placed a ±25 µm thick transparent polyester strip (TDV, Pomerode, SC, Brazil) and a microscope slide followed by manual pressure for 15 s to adapt the resin cement to the mold and to remove the cement excess. The microscope slide was removed, and a ceramic disc was placed on the polyester strip. The underlying resin cement was exposed to light curing through the ceramic for 40 s using the Valo photocuring light with an irradiance of 834 mW/cm^2^. During light activation, the light guide (diameter of 9.75) was centered in contact with the glaze ceramic surface. For the control group, the light guide end was in contact with the polyester strip. All the procedures were performed in room under conditions of humidity (50 ± 10%) and temperature (23 ± 2°C) with controlled ambient filtered red-light in order to prevent photoinitiator sensitization. After light-curing, the resin cement samples were stored dry in the dark at 37 °C for 24 h.

To obtain a flat and polished surface for the resin cement KHN measurements, the resin cement was attached to an acrylic resin plate with sticky wax (Buhler, Lake Bluff, IL, USA), fixed, transversely wet-cut using a saw, and polished using 320-, 400-, 600-, and 1200-grit silicon-carbide abrasive papers (Norton; São Paulo, SP, Brazil) in a water-cooled automatic polisher (APL4; Arotec, Cotia, SP, Brazil).

Knoop microhardness measurements were performed (HMV-2; Shimadzu, Tokyo, Japan) under a load of 50 g applied for 15 s. Five indentations were made across depths of 100 and 700 µm from the top surface. The average value of the five readings was recorded as the Knoop hardness for that specific sample at the given depth.

Knoop microhardness data after the exploratory analysis were submitted to analysis of variance (ANOVA) in a split plot design, with the parcels represented by the factorial 3 × 3 × 3 (translucency × shade × thickness) and the sub-parcels by layer (top and bottom), with two additional control treatments. Appropriate post-hoc pair wise comparisons were made using Tukey’s test (SAS Institute Inc., Cary, NC, USA, version 9.3). All statistical tests were performed at *α* = 0.05.

## Results

### Transmitted irradiance (mW/cm²)

The mean values for transmitted irradiance are given in Table 1. Significant influences of translucency (*p* < 0.0001), shade (*p* < 0.001), and thickness (*p* < 0.0001) were detected. The interaction between translucency and shade (*p* < 0.0001), translucency and thickness (*p* < 0.0001), and shade and thickness (*p* = 0.0018) showed significant values. The triple interaction translucency vs shade vs thickness was significant (*p* = 0.0052).

**Table 1:** Mean (standard deviation) for transmitted irradiance (mW/cm^2^) through the ceramic as a function of ceramic thickness, shade and translucency

Thickness (mm)	Shade	Translucency		
		HT	LT	MO
0.5	A3.5	^a,b,c^635.15 (13.22) Aa	^a,b,c^610.87 (8.07) Aa	^a,b,c^512.36 (19.38) Ba
	A1	^a,b,c^614.88 (8.94) Aa	^a,b,c^550.99 (12.26) Bb	^a,b,c^501.41 (15.84) Ca
	BL2	^a,b,c^554.16 (19.06) Ab	^a,b,c^527.83 (13.63) Ab	^a,b,c^474.51 (1.61) Ba
1.5	A3.5	^a^468.46 (43.45) Aab	^a,c^457.51 (35.81) Aa	^a^391.41 (53.38) Ba
	A1	^a^503.62 (28.43) Aa	^a,c^436.75 (32.27) Ba	^a^356.24 (13.11) Ca
	BL2	^a^432.91 (12.76) Ab	^a^402.84 (4.22) ABa	^a^334.72 (7.29) Ba
2.0	A3.5	^a^435.79 (31.41) Aab	^a^356.72 (64.83) Ba	^a^366.33 (33.30) Ba
	A1	^a^480.18 (15.33) Aa	^a^335.68 (32.10) Ba	^a^354.42 (6.32) Ba
	BL2	^a^375.07 (8.26) Ab	^a^334.72 (16.01) ABa	^a^288.81 (7.80) Bb

Control group: mean (standard deviation): 834.22 (8.78). Means followed by distinct letters (upper-case horizontal and lower-case vertical comparing shade in each thickness and translucency) differ from each other (*p* < 0.05)

*HT* high translucency, *LT* low translucency, *MO* medium opacity

^a^Differs from the control group (*p* ≤ 0.05)

^b^Differs from thickness 1.5 under the same conditions of translucency and shade (*p* ≤ 0.05)

^c^Differs from thickness 2.0 under the same conditions of translucency and shade (*p* ≤ 0.05)

When ceramic translucency was compared, the irradiance of HT was significantly greater than MO for all shades and thicknesses. HT showed higher irradiance values than LT for shade A1 in all thicknesses, and for shades A3.5 at thickness 2.0 mm (*p* < 0.05). No significant difference in irradiance was observed between ceramic translucency for LT and MO (*p* > 0.05), except shade A3.5 and A1 for thickness 1.5 mm, and for all shades and thickness 0.5 mm (*p* < 0.05).

When shades were compared for HT, the irradiance for shades A3.5 and A1 were significantly higher than BL2 for thickness 0.5 mm (*p* < 0.05), and A1 was significantly higher than BL2 for thicknesses 1.5 mm and 2.0 mm (*p* < 0.05). No difference was found between A1 and A3.5 for thicknesses 1.5 mm and 2.0 mm. For LT, shade A3.5 and thickness 0.5 mm was significantly higher than shades A1 and BL2 (*p* < 0.05). No significant difference was found among shades for thicknesses 1.5 mm and 2.0 mm (*p* > 0.05). No difference was observed for MO in any shade and thicknesses 0.5 mm and 1.5 mm. Shade BL2 for thickness 2.0 mm showed lower irradiance compared to the two other shades, but no significant difference was found between shades A3.5 and A1. (*p* > 0.05).

The control group demonstrated statistically higher irradiance compared to all ceramic shades, translucencies and thicknesses (*p* < 0.05). For all shades, HT and MO translucencies, there was significant reduction in irradiance for thicknesses 1.5 mm and 2.0 mm when compared to 0.5 mm thickness (*p* < 0.05). No significant difference was found between thicknesses 1.5 mm and 2.0 mm (*p* > 0.05). When LT was analyzed, the irradiance for thickness 0.5 mm was significantly greater than thickness 2.0 for all shades (*p* < 0.05). For thickness 1.5 mm, shades A3.5 and A1 were significantly higher than shade BL2 and for the three shades with 2.0 mm ceramic thickness (*p* < 0.05).

### Knoop microhardness test

The mean values for Knoop microhardness are summarized in Table 2. Significant influence of translucency (*p* < 0.0001), shade (*p* < 0.001), thickness (*p* < 0.0001), and depth (*p* < 0.0001) was detected. The double interaction between translucency vs shade (*p* < 0001), translucency vs depth (*p* = 0.0148), and thickness vs depth (*p* = 0.0004) values show significant interaction of factors. No significant interaction of factors was found for translucency vs thickness (*p* = 0.1858), shade vs thickness (*p* = 0.0950), and shade vs depth (*p* = 0.0840). The triple interaction translucency vs shade vs depth (*p* = 0.0497) and translucency vs thickness vs depth (*p* < 0.0001) was significant. Translucency vs shade vs thickness, and shade vs thickness vs depth (*p* < 0.0940) did not show significant interaction. The quadruple interaction translucency vs shade vs thickness vs depth (*p* = 0.2827) was not significant.

**Table 2:** Mean (standard deviation) of resin cement Knoop hardness as a function of ceramic translucency; shade; thickness; and depth into the cement

Depth (μm)	Thickness (mm)	Shade	Translucency		
			HT	LT	MO
100	0.5	A3.5	^abc^30.34 (1.40) Aa	^abcd^24.28 (1.49) Ba	^abcd^15.62 (1.32) Ca
		A1	^abcd^27.40 (1.69) Ab	^abcd^24.52 (1.23) Ba	^abcd^13.14 (1.16) Cab
		BL2	^abcd^28.12 (2.36) Ab	^abcd^20.44 (0.64) Bb	^abcd^12.09 (0.77) Cb
	1.5	A3.5	^acd^25.53 (1.13) Aa	^acd^20.36 (1.11) Ba	^acd^11.31 (0.85) Ca
		A1	^acd^23.61 (1.45) Ab	^acd^20.90 (0.58) Ba	^acd^10.97 (1.00) Cab
		BL2	^acd^23.12 (1.33) Ab	^acd^17.90 (0.90) Bb	^acd^10.39 (0.75) Cb
	2.0	A3.5	^ad^23.85 (1.13) Aa	^ad^17.82 (0.39) Ba	^ad^9.19 (0.77) Ca
		A1	^ad^20.35 (1.37) Ab	^ad^17.30 (0.95) Ba	^ad^9.07 (1.38) Cab
		BL2	^ad^20.70 (0.78) Ab	^ad^15.39 (0.89) Bb	^ad^7.58 (0.71) Cb
700	0.5	A3.5	^bcd^24.24 (1.02) Aa	^bcd^19.57 (1.03) Ba	^bcd^10.73 (1.45) Ca
		A1	^bcd^23.14 (1.17) Ab	^bcd^19.47 (1.23) Ba	^bcd^9.5 6 (0.59) Ca
		BL2	^bcd^22.59 (2.25) Ab	^bcd^15.86 (0.92) Bb	^bcd^8.28 (1.14) Ca
	1.5	A3.5	^d^20.72 (1.10) Aa	^d^15.98 (1.12) Ba	^d^5.60 (1.07) Ca
		A1	^dc^18.71 (0.69) Ab	^dc^16.40 (0.96) Ba	^dc^6.30 (1.45) Ca
		BL2	^dc^18.69 (1.72) Ab	^dc^13.78 (0.92) Bb	^dc^5.90 (0.82) Ca
	2.0	A3.5	^d^20.28 (0.99) Aa	^d^14.73 (0.40) Ba	^d^4.06 (0.49) Ca
		A1	^d^16.75 (0.57) Ab	^d^14.16 (0.45) Ba	^d^3.73 (0.56) Ca
		BL2	^d^16.11 (0.86) Ab	^d^11.77 (0.63) Bb	^d^3.37 (0.37) Ca

Control group: mean (standard deviation): 100 μm − 30.42 (0.75) / 700 μm − 28.01 (1.68). Means followed by distinct letters (upper-case horizontal and lower-case vertical comparing shade within each thickness and translucency) differ from each other (*p* ≤ 0.05)

*HT* high translucency, *LT* low translucency, *MO* medium opacity

^a^Differs from 700 μm under the same conditions of translucency, shade and thickness (*p* ≤ 0.05)

^b^Differs from thickness 1.5 under the same conditions of translucency, shade and location (*p* ≤ 0.05)

^c^Differs from thickness 2.0 under the same conditions of translucency, shade and location (*p* ≤ 0.05)

^d^Differs from the control group (*p* ≤ 0.05)

When ceramic opacity/translucency was compared, the mean Knoop microhardness for HT was significantly higher than LT and MO regardless of shade, thickness and depth (*p* < 0.05). When shades were compared for HT, the mean Knoop hardness for A3.5 was significantly higher in relation to A1 and BL2 (*p* < 0.05) for all thicknesses and depths. For LT, A3.5 and A1 were significantly higher than BL2 (*p* < 0.05) for all thicknesses and depths. With relation to MO, A3.5 was significantly higher than BL2 (*p* < 0.05) for all thicknesses at the 100 μm depth. Shade A1 did not differ statistically from the A3.5 and BL2. No significant difference was found among shades for all thicknesses at the 700 μm depth (*p* > 0.05).

The control group demonstrated significantly higher Knoop microhardness in relation to all ceramic shades, translucencies and thicknesses (*p* < 0.05), except for HT, shade A3.5, thickness 0.5 mm at 100 μm depth. Thickness 0.5 mm was statically significantly higher than 1.5 mm (*p* > 0.05), regardless of translucency, shade and depth. For all shades, translucency and depth showed significant reduction in microhardness for thickness 2.0 mm in relation to 0.5 mm and 1.5 mm thicknesses (*p* < 0.05), except for the 700 μm depth for all translucencies of shade A3.5, 1.5 mm thickness. For all shades, thicknesses and translucencies there was significant reduction in microhardness at 700 μm depth compared to 100 μm (*p* < 0.05).

## Discussion

The first hypothesis, which stated that transmitted irradiance is not impacted among the different shades, translucencies and thicknesses of the ceramic was rejected. The results showed that the irradiance values (mW/cm^2^) were affected through different ceramic shades, translucencies and thicknesses. The second hypothesis was also rejected, Knoop hardness of the resin cement was impacted by different ceramic shades, translucencies, shades, thicknesses and depth.

A minimum irradiance of 400 mW/cm² with wavelengths between 400 nm and 500 nm (Blue light range) for 40 s is thought adequate to initiate the activation of camphorquinone initiated resin materials [24, 25]. The Ivocerin used in Variolink Esthetic is photo-activated by wavelengths between 380 nm and 400 nm (violet – blue range) [16, 26] and according to the manufacturer’s recommendations (Ivoclar Vivadent, Schaan, Liechtenstein), a light exposure of 500–1000 mW/cm^2^ for 20 s is required to cure through a 1 mm ceramic restoration.

In the present study, the transmitted irradiance (mW/cm²) was under 500 mW/cm^2^ for most of the groups, except for HT 0.5 mm (all shades) and 1.5 mm shade A1; for LT 0.5 mm (all shades); and for MO 0.5 mm shades A3.5 and A1. Generally, lower irradiance values were transmitted through lithium disilicate with decreased ceramic translucency, increased thickness and more opaque shade (BL2) (Table 1). In addition, the irradiance values for the control group (834.22 ± 8.78 mW/cm^2^) was higher than by the interposition of ceramic discs, regardless of factors. This was expected and consistent with previous studies that indicated these results might be due to absorption or refraction of the light [27, 28]. Thus, the transmitted irradiance and an adequate polymerization of underlying resin cement are correlated [21], since the light attenuation promoted by interposition of a ceramic material compromised the resin cement mechanical properties [29].

Hardness measurements are commonly used as a simple method that indirectly correlates the degree of conversion and polymerization efficiency of resin-based materials [30–35]. A study by Calgaro and others [30] showed a positive correlation between the degree of conversion and Knoop Hardness of resin cements affected by ceramic interposition. In the present study, Knoop microhardness of the resin cement was affected by the translucency of the ceramic material. There was an increased microhardness with increased translucency as follows: Medium Opacity < Low Translucency < High Translucency, regardless of shade, thickness or depth (Table 2). These findings are in agreement with previous studies, which also found reduced hardness values when ceramic opacity was increased [29, 36, 37]. This was not a surprising result because more translucent ceramics allowed higher light transmission and, consequently, the total energy received was greater than for less translucent ceramic. Previous studies also showed that the translucency of ceramics can be affected by several factors, such as microstructure, texture, thickness, illuminant, number of firings, shade and luting agent [22, 23, 38–41].

A strong relationship between translucency and thickness was previously reported. Wang and others [38] showed that the translucency of different types of ceramic decreased exponentially as the thickness increased. The highly translucent ceramic would be significantly affected by thickness variations, therefore, both factors should be considered to achieve the best esthetic outcomes clinically [42].

In relation to the factor thickness, the results of the present study showed that Knoop microhardness increased as the thickness decreased, and it was also affected by depth within the cement and ceramic shade, regardless of translucency. These results are consistent with previous studies which showed that when the thickness of the ceramic increased, a reduction of the irradiance and total energy was observed [21, 43]. No statistical difference was found between 1.5 mm and 2.0 mm for shade A3.5 at 700 μm depth for all translucencies. This might be explained by the fact that shade A3.5 allowed greater light transmission to the luting agent. Other studies showed that ceramic thickness and translucency parameters have a greater effect on the polymerization than ceramic shade [21–29, 36–39].

When the Knoop microhardness was analyzed according to ceramic shade, statistically significant differences between shades were shown between different translucencies and depths. In this study, three ceramic translucencies of different shades were evaluated, ranging from higher opacity (BL2), corresponding to an opaque white bleach shade, to low opacity and ranked: BL2 > A3.5 > A1. Others have demonstrated that less light reaches the resin cement when darker shades (higher chroma) are used, since the dark pigments absorb light and negatively influence the polymerization of the resin cement under a restoration [17, 22]. The results of the current study are partially consistent with those reports, since the lowest microhardness was found for the opaque shade (BL2) in LT for both depths, all thicknesses and at 100 μm for MO, regardless of thickness. In addition, no difference was found between shade A1 and BL2 for HT and between A3.5 and A1 for LT. These results further support the fact that no difference in cement microhardness was found when using different ceramic shades, suggesting that the microhardness is more dependent upon translucency than upon shade [23, 44]. However, the opposite effect was also observed in the current study, since shade A3.5, which has higher chroma than shade A1, allowed greater light transmission and higher microhardness values for HT. These findings are in agreement with another study that observed higher %DC for ceramic shade 0M2 (higher chroma) than for shade 0M1 (lower chroma) when a transparent resin cement shade was photocured for 40 s [22]. Our results might be explained by the specific interaction of factors studied that interfered in the polymerization of underlying resin cement. Thus, an understanding of varying these factors to achieve the best clinical performance is needed.

For the depth factor, significant reduction in the Knoop microhardness was observed at the depth of 700 μm compared to 100 μm. These findings are in agreement with previous studies, which also found higher values at the top 100 µm compared to 700 µm [12, 45, 46]. Previous studies also showed that the incident light was attenuated as the distance from the irradiated surface increases. This fact occurs due to the absorption and scattering promoted by the fillers and resin components, reducing the polymerization effectiveness as the depth increases [27, 47]. Another study showed that only 25% of light incident on the surface of a resin composite is observed at 1 mm depth [27]. It is important to recognize that a cement thickness of 700 μm is not used for clinical luting purposes and that light attenuation induced by indirect ceramic restorations could be worsened by low irradiance levels of the light curing device [17, 43].

In the present study, the control group (without ceramic interposition) demonstrated significantly higher Knoop microhardness compared to the ceramic groups, except for HT, shade A3.5 and 0.5 mm of thickness at 100 μm depth where no difference was found. This may be explained by the fact that these factors showed the highest values of microhardness when they were ranked separately: HT > LT > MO (translucency); 0.5 mm > 1.5 mm > 2.0 mm (thickness); A3.5 ≥ A1 > BL2 (shade); and, 100 μm > 700 μm (depth).

In summary, shade, translucency, and thickness of a pressable lithium disilicate ceramic have significant impact on the transmitted irradiance and Knoop microhardness of the resin cement at different depths. Incomplete polymerization of the resin cement may decrease mechanical properties and increase water sorption of the resin cement. Care should be taken in clinical practice with increased ceramic opacity, thickness and more opaque shades because the reduction of light transmission may negatively impact the resin cement.

It is important to note that the data discussed in this study were limited to the assessment of one physical behavior of the resin cement (hardness) in a single shade, subjected to light cure through a lithium disilicate ceramic. Further studies are needed to analyze the impact of these associated factors on the cement bond strength as well as the influence of different shades of the resin cement, LCU devices, light intensities, and exposure times to gain further insight into these clinically important parameters. In addition, actual clinical studies manipulating these variables would help clarify the clinical relevance of these laboratory findings.

## Conclusions

Based on these findings, the following conclusions may be drawn:

- Different shades, translucencies and thicknesses of pressable lithium disilicate have a significant effect on transmitted irradiance.
- Interposition of pressable lithium disilicate and increased thicknesses of ceramic significantly reduce the Knoop microhardness of underlying resin cement.
- Microhardness of resin cement at 700 μm depth is significantly reduced for all translucencies, thicknesses, and shades of overlying pressable lithium disilicate.
- Increased pressable lithium disilicate opacity reduces cement microhardness values at all depths regardless of ceramic thickness or shade.
